# CohAlign: Predicting the first-order noise susceptibility of gradients in equivariant quantum neural networks

**DOI:** 10.1371/journal.pone.0361139

**Published:** 2026-10-22

**Authors:** Hassan Ugail, Newton Howard

**Affiliations:** 1 Centre for Visual Computing and Intelligent Systems, University of Bradford, Bradford, United Kingdom; 2 School of Individualized Study, Rochester Institute of Technology, Rochester, New York, United States of America; Commonwealth Scientific and Industrial Research Organisation, AUSTRALIA

## Abstract

Quantum neural networks learn by following gradients, and on real hardware those gradients are washed out by noise. Predicting how quickly this happens, before any finite noise experiment or circuit sweep is run, remains an open problem. We present CohAlign, an open source toolkit that computes the first order noise susceptibility of the ensemble second moment of a specified parameter gradient in a charge conserving brickwork quantum neural network. The computation needs only a classical description of the circuit, the noise model, the prepared state, the readout and the initialisation prior. The predicted quantity is distinct from training convergence or final accuracy. The key observation is that only the part of the noise that a measurement can actually see matters. A purposefully constructed control channel makes this clear, damaging the operator carrying a gradient at nearly the maximal rate while the measured gradient remains exactly as predicted and untouched. We validate the predictions against separately evaluated finite difference slopes across the benchmark suite, including a signed coherent case, in exact simulations at eight and ten qubits and on two circuit topologies.

## 1. Introduction

Variational quantum algorithms are among the leading candidates for useful computation on near term quantum hardware. The idea is simple. A circuit carries trainable parameters, a cost function is measured at its output, and the parameters are adjusted until the measured cost falls [1,2], with gradients obtained through parameter-shift rules [3–5]. The central obstacle to scaling this approach is trainability. Deep or highly expressive circuits suffer from barren plateaus, regions of the landscape where gradients vanish exponentially with system size [6–8], and a growing body of theory identifies architectures and initialisations that avoid them [9–11]. Hardware noise, however, introduces a second and quite distinct mechanism. Even when the noiseless landscape is benign, noise accumulating through the layers suppresses the measured gradients, in the worst case producing noise induced plateaus that no initialisation can repair [12–14]. This second mechanism, and the problem of predicting it before any experiment is run, is what the present paper addresses.

Symmetry offers a promising route through both difficulties. Equivariant architectures restrict every gate to commute with a conserved charge and are among the most attractive routes to trainable models [15–20], and for our purposes the symmetry brings a further and less widely appreciated benefit. Because the dynamics is confined to a fixed charge sector, the cost function and its gradients live in a known part of the Hilbert space, and only a specific portion of the coherence within that sector is visible to a local readout. A single scalar summary of the noise therefore paints too pessimistic a picture, because it says nothing about where the damage falls. The central contrast of this paper is that channel contraction is not the measured gradient degradation. Two channels with identical worst case rates can degrade gradients at very different speeds, and a channel that rapidly contracts almost every coherence direction can leave the gradients entirely untouched, provided the directions it contracts are the ones the readout cannot see.

This paper makes that observation quantitative and turns it into a working software instrument. The setting is the family of brickwork quantum neural networks conserving a global charge under phase rotations. The central mathematical object is the in-sector off-diagonal block of the noise generator, the occupation basis coherences within a degenerate charge eigenspace, which constitute the sector relative form of quantum coherence [21–23]. In the mode decomposition of Marvian and Spekkens [24,25] these coherences belong to the zero frequency mode of the global phase action, generated by the degeneracy of the sector. They are therefore invariant under the symmetry rather than resources of asymmetry, and this is precisely why the covariance of a channel with the symmetry offers them no protection.

On this block it is helpful to distinguish three quantities, each answering a progressively sharper question. The worst case sector coherence rate $\lambda_{coh}$ measures the fastest speed at which the channel contracts any coherence direction, and so tells us how bad the noise could possibly be. The operator mode contraction rate $\lambda_{mode}$ measures how fast the channel contracts the particular operator mode carrying the derivative of the readout with respect to a chosen parameter, and so tells us how badly the noise damages the object that carries the gradient. The response susceptibility $\Lambda^{resp}$ measures how fast the initialisation ensemble second moment of the measured gradient decays, and depends jointly on the channel, the architecture, the parameter, the readout, the prepared state and the initialisation ensemble.

The progression from $\lambda_{coh}$ through $\lambda_{mode}$ to $\Lambda^{resp}$ is the organising spine of the paper, each step conditioning the question on more of the problem, and a central lesson is that the last two are genuinely different objects. We exhibit a case in which the channel contracts the gradient mode at almost the full worst case rate while the measured gradient does not decay at all, simply because the contraction is invisible to the readout. This is the direct answer to the natural question of why an operator contraction rate is not already sufficient.

The practical contribution is a toolkit, called CohAlign, that computes all three quantities ahead of time from a classical description of the problem, with no finite noise circuit sweep and no measured degradation data. It does require a classical simulation of the noiseless circuit at the system sizes we study, so we describe the method throughout as a classical design time calculation. The toolkit is validated on a ladder of tests of increasing difficulty. The ladder begins with an exact analytic recovery and an engineered control whose gradient is provably immune to its noise, continues through a direct finite difference validation of the susceptibility itself, extended across parameter locations, a second circuit topology, a larger system and varied settings. The ladder ends with a family wide comparison across nine noise models and a study in the two excitation sector, where scalar descriptions are least likely to suffice.

This paper is standalone in the sense that every definition, derivation, dataset and line of code needed to reproduce its results is contained in the paper and its released pipeline. No result here depends on our earlier noiseless symmetry audits [26,27] or channel level diagnostics [28], which do not address the parameter resolved, readout aware prediction problem treated here.

The remainder of the paper is organised as follows. Section 2 defines the model, the three rates and the validation protocol, Section 3 presents the validation ladder, Section 4 interprets the findings and states the limitations, and Section 5 concludes, with two appendices collecting the ensemble derivation, the test battery and a worked example.

## 2. Materials and methods

### 2.1. Ethics statement

This study did not involve human participants, animal experimentation or personal data. All experiments are numerical simulations implemented in open source scientific Python libraries, and no institutional ethics approval was required.

### 2.2. The model, the readout and the noise channels

The architecture we study is a brickwork quantum neural network on a ring of *n* qubits, and the implementation also provides an open chain variant, which drops the cycle closing edge, together with a selectable readout site, both used in the validation reported below. Each layer applies a trainable phase rotation $R_{z}(\theta_{\ell q})=\exp(-i\theta_{\ell q}Z_{q}/2)$ on every site, followed by a fixed layer of hopping gates $e^{-i\beta_{\ell j}(X_{j}X_{j+1}+Y_{j}Y_{j+1})}$ on alternating edge sets, even numbered edges in even layers and odd numbered edges in odd layers, with the ring closing edge included. All indices are zero based. Every gate commutes with the total charge $\hat{N}=\sum_{q}(I-Z_{q})/2$, so the dynamics never mixes charge sectors.

The readout is the sector projected observable $O_{B}=P_{r}Z_{0}P_{r}$, where $P_{r}$ projects onto the sector with *r* excitations, and the input is a fixed state in that sector, in the single excitation case the basis state with its excitation on site zero. The hopping angles are drawn once from a seeded uniform distribution on [−0.5,0.5] with seed 42 and frozen, and the trainable phases are drawn from a small box of width $\delta_{init}=0.05$ around zero, the small angle regime commonly used to avoid noiseless plateaus [11].

Noise enters the model in a controllable way. After every layer, a noise channel $\Phi_{\gamma }$ acts once on the full register, where γ is the per layer strength, and because a coherence rate depends on the convention linking γ to the elementary error probabilities, we state each convention explicitly. Four channels act identically on every site through a single qubit Kraus pair. Amplitude damping uses the standard pair with damping probability γ, dephasing applies the phase flip *Z* with probability γ, depolarising applies each Pauli with probability γ/3, and the *X* error channel applies the bit flip with probability γ. We call these the restricted isotropic family, since their restricted generators contract every direction of the sector coherence block at a common rate. Two structured variants, inhomogeneous dephasing and site dependent amplitude damping, multiply the per site probability by a fixed linear profile rising from 0.5 to 1.5 across the ring, normalised to unit mean. Biased Pauli noise distributes the total probability γ with the tuple $\left(p_{X},p_{Y},p_{Z})=(0.6,0.2,0.2\right)$, deliberately breaking phase covariance because $p_{X}$ and $p_{Y}$ differ.

The last two channels are controls in the strict sense, designed so that the correct answer is known before any computation is run. The coherent dissipative channel applies a uniform coherent *Z* rotation of angle 0.5γ on every site followed by amplitude damping at γ. A uniform rotation of this kind is generated by an operator proportional to the conserved charge, so within any fixed sector it acts as a global phase with no operational effect. The audit must therefore return results identical to plain amplitude damping. The results confirm this exactly, and a genuinely active coherent perturbation is exercised separately through the signed demonstration below. The correlated dephasing control applies the two site phase flip Z⊗Z with probability γ on each edge of the disjoint pairing of even edges, composed sequentially. Coherence within a paired edge is therefore exactly preserved while coherence across edges decays quickly, precisely the structure that lets a large worst case rate coexist with an invisible effect on particular gradients.

It is useful to record which of these channels respect the symmetry of the architecture. A channel is phase covariant when it commutes with the charge rotations, which we write as


$$\begin{array}{c} \Phi \;\left(U_{\phi }\rho \;U_{\phi }^{†}\right)=U_{\phi }\;\Phi (\rho )\;U_{\phi }^{†},\;U_{\phi }=e^{-i\phi \hat{N}}, \end{array}$$ (1)

for every angle ϕ, a class with a classical structure theory [29]. Covariance is independent of sector retention. The dephasing type channels and the correlated control keep every sector population fixed, whereas the damping channels move population downwards between sectors and the Pauli type channels transfer it in both directions even when covariant. The projected construction of the next subsection handles both cases identically, since any output component leaving the off-diagonal sector block is discarded, and for sector changing channels that discarded component is the leakage contribution.

Thus, six channels are phase covariant with purely dissipative generators, the *X* error and biased Pauli channels break covariance and serve as leakage probes, and the coherent dissipative channel is the null coherent control. The released pipeline additionally demonstrates the audit on a pure coherent perturbation, a site dependent *Z* over-rotation whose phase profile is not proportional to the conserved charge and therefore acts non-trivially on the sector coherences. For such a generator, the worst case rate is numerically zero, the response is a signed quantity, and the software reports the signed value with an explicit flag instead of any normalised ratio.

### 2.3. Generator level rates and the operator mode contraction diagnostic

Having fixed the circuit, the state and the noise, we can now ask how quickly a given channel destroys the coherence on which the network relies. Here we describe how the channel is converted into the reported rates, and Table 1 collects the recurring symbols in one place.

**Table 1:** Notation used throughout.

Symbol	Meaning
γ, γL	per layer noise strength and accumulated strength over *L* layers
$\gamma_{probe}$	finite probe strength used to extract the restricted generator
${ℒ}_{r}$	restricted generator on the charge sector coherence block
$\lambda_{coh}$, $\lambda_{typ}$	worst case and typical sector coherence contraction rates
$\lambda_{mode}$	operator mode contraction rate of the gradient carrying mode
$\Lambda^{resp}$	response susceptibility of the ensemble gradient second moment
$\omega^{resp}$, $\hat{\omega }$	predicted alignment ratio and its measured small noise intercept
$\tilde{\Delta }$	measured relative degradation of the gradient second moment

One convention should be stated at the outset. The projector $\Pi_{off,r}$, the rate $\lambda_{coh}$ and the coherence language throughout are defined relative to the occupation number basis of the sector, the computational basis restricted to it, and they are not invariant under arbitrary basis changes inside the sector. This choice is matched to the diagonal readout and the dephasing dominated channel families studied here. Writing $\Phi_{\gamma }=id+\gamma ℒ+O(\gamma^{2})$, the object of interest is the Markovian generator ℒ [30,31], with each channel specified through its Kraus operators [32,33]. The toolkit restricts the generator to the off-diagonal part of the chosen sector.

Let the pair basis consist of the operators |a⟩⟨b| with *a* and *b* distinct elements of the sector basis, let *V* embed pair basis coefficient vectors into operators, and let $\Pi_{off,r}$ project onto the span of the pair basis. The restricted superoperator is then the projected map


$$\begin{array}{c} S_{\gamma_{probe}}\;=\;V^{†}\;\Pi_{off,r}\;\Phi_{\gamma_{probe}}\;\Pi_{off,r}\;V,\;{ℒ}_{r}\;=\;\frac{S_{\gamma_{probe}}-I}{\gamma_{probe}}, \end{array}$$ (2)

built column by column at the probe strength $\gamma_{probe}=10^{-3}$. Any output component outside the off-diagonal block is discarded, which is how sector leakage enters for symmetry breaking channels, and the finite difference carries a bias of order $\gamma_{probe}$ to which we return below.

The worst case sector coherence rate is the variational supremum of the decay Rayleigh quotient over the coherence block,


$$\begin{array}{c} \lambda_{coh}\;=\;\underset{A\neq 0}{\sup}\frac{-\;Re\;\langle A,{ℒ}_{r}A\rangle }{\langle A,A\rangle }\;=\;\max eig\;\left[-\frac{1}{2}\left({ℒ}_{r}+{ℒ}_{r}^{†}\right)\right], \end{array}$$ (3)

computed spectrally in the Frobenius geometry, with the typical rate the mean of the same spectrum. This construction is finer than scalar whole channel summaries such as the average gate fidelity [34] or the unitarity of the noise [35], which characterise the channel independently of any architecture or readout. The rates here instead resolve what the channel does to the specific coherence a given measurement can see.

For the four reference isotropic channels, the finite probe values of $\lambda_{coh}$ tend towards the limits 1, 4, 20/3 and 8 as the probe is reduced, linearly in the probe exactly as the construction predicts. Table 2 makes the sensitivity to the probe choice explicit. Recovery statements are therefore made as relative tolerances at the working probe, and isotropy is certified by the spectral spread of the Hermitian part of the restricted generator.

**Table 2:** Sensitivity of the worst case rate to the probe strength. Finite probe values of $\lambda_{coh}$ for the four reference isotropic channels, converging linearly to the analytic limits 1, 4, 20/3 and 8 as the probe is reduced. Amplitude damping is reproduced exactly at every probe because its restricted generator is linear in the strength. The shipped working probe is 10^–3^.

$\gamma_{probe}$	Amplitude damping	Dephasing	Depolarising	*X* error
10^–2^	1.000	3.9600	6.4786	7.7349
10^–3^	1.000	3.9960	6.6476	7.9730
10^–4^	1.000	3.9996	6.6648	7.9973
10^–5^	1.000	3.99996	6.6665	7.9997

The first architecture aware quantity is the operator mode contraction rate. For a parameter at layer $\ell_{i}$ and site $q_{i}$, the Heisenberg readout at the insertion point is obtained by evolving $O_{B}$ backwards through all later layers and through the hopping block of layer $\ell_{i}$ itself. The rotation sits before its own layer’s hopping gates, and omitting that block silently deactivates the final layer. The gradient mode is the projected commutator


$$\begin{array}{c} G_{i}(\theta )\;=\;\Pi_{off,r}\left[Z_{q_{i}},\;O_{B}(ins;\theta )\right], \end{array}$$ (4)

which is carried forward to each noise slot at or after the insertion layer. The mode contraction rate is the small box ensemble average of the decay Rayleigh quotient of the restricted generator on these slot modes,


$$\begin{array}{c} \lambda_{mode}\;=\;{𝔼}_{\theta }\;{\overset{―}{\left[\frac{-\;Re\;\langle G_{i}^{\left(\ell \right)},{ℒ}_{r}\;G_{i}^{\left(\ell \right)}\rangle }{\langle G_{i}^{\left(\ell \right)},G_{i}^{\left(\ell \right)}\rangle }\right]}}_{\ell }, \end{array}$$ (5)

where the overline is the mean over noise slots. Once the channel, architecture, parameter and prior are fixed, this quantity is independent of the prepared state. It answers how fast the channel contracts the gradient carrying operator. That is not the operational question of how fast the measured gradient decays, since the decay also depends on how the contracted operator is read out through the prepared state, and the results show the two answers can differ by nearly the full worst case rate.

### 2.4. The response susceptibility of the measured gradient

The rates of the previous subsection describe what the noise does to operators, but what matters for training is what the noise does to the gradient that is actually measured, and the two need not agree. The central quantity of the paper therefore asks directly how the derivative of the measured cost responds to a small amount of noise at each slot. Let $g_{0}(\theta )$ be the noiseless derivative at the draw θ. Expanding the noisy derivative to first order gives


$$\begin{array}{c} g(\gamma ,\theta )\;=\;g_{0}(\theta )\;+\;\gamma \sum_{\ell }h_{\ell }(\theta )\;+\;O(\gamma^{2}), \end{array}$$ (6)

in which $h_{\ell }(\theta )$ is the first order response of the derivative to a generator insertion at slot ℓ. The response terms have closed forms evaluated exactly within the finite probe approximation. For a slot at or after the insertion layer the term is $h_{\ell }=Re\;Tr(O_{B}^{\left(\ell \right)}ℒ(D^{\left(\ell \right)}))$, with $D^{\left(\ell \right)}$ the derivative operator propagated forwards and $O_{B}^{\left(\ell \right)}$ the readout evolved backwards. For an earlier slot it is $h_{\ell }=Re\;Tr(F^{\left(\ell \right)}ℒ(\rho_{\ell }))$, with $\rho_{\ell }$ the noiseless state and $F^{\left(\ell \right)}$ the adjoint response functional. The noiseless pairing reproduces the derivative itself at every slot to better than one part in 10^8^ in the shipped tests.

The quantity that governs the measured degradation is a cross moment of the noiseless derivative with its response. The measured second moment of the gradient over the initialisation ensemble is $M_{2}(\gamma )={𝔼}_{\theta }[\;g(\gamma ,\theta {)}^{2}\;]$, and inserting the expansion of Equation (6) gives


$$\begin{array}{c} \frac{M_{2}(\gamma )}{M_{2}(0)}\;=\;1\;-\;2\gamma \;\Lambda^{resp}\;+\;O(\gamma^{2}),\;\Lambda^{resp}\;=\;-\;\sum_{\ell }\frac{{𝔼}_{\theta }\;\left[g_{0}(\theta )\;h_{\ell }(\theta )\right]}{{𝔼}_{\theta }\;\left[g_{0}(\theta {)}^{2}\right]}. \end{array}$$ (7)

This cross moment form is the definition the toolkit implements directly. The estimator accumulates the numerator and denominator of Equation (7) separately over the draws and never divides by an individual draw, so it remains defined when a draw has a vanishing derivative, every sampled draw contributes, and no derivative threshold enters the prediction. Per draw ratios $r_{\ell }(\theta )=-h_{\ell }(\theta )/g_{0}(\theta )$ are reported only as an optional diagnostic for well resolved draws.

Two ensembles appear in the study, an estimator ensemble of forty draws and a separate common random number measurement ensemble of thirty draws, each with its own recorded seed. A floor of 10^–12^ on the squared derivative guards the measurement side, below which no draw fell in any headline configuration, the marginal locations of the parameter resolved map being the recorded exception. The cross moment admits a standard bootstrap for a ratio of sample means [36], and Appendix A derives Equation (7) and explains why a plain mean of per draw ratios becomes unstable in deep circuits. The toolkit also reports a weight concentration diagnostic $(\sum w{)}^{2}/\sum w^{2}$ with $w=g_{0}^{2}$, indicating how many draws effectively carry the estimate.

From the response susceptibility the toolkit forms the first order degradation prediction and, for the contractive channels, a normalised alignment ratio,


$$\begin{array}{c} {\tilde{\Delta }}_{pred}\;=\;2\gamma \;\Lambda^{resp},\;\omega^{resp}\;=\;\frac{\Lambda^{resp}}{L\;\lambda_{coh}}, \end{array}$$ (8)

which is directly comparable to the empirical ratio $\hat{\omega }=\tilde{\Delta }/(2\gamma L\lambda_{coh})$ from paired measurements. A word of caution attaches to the normalised form. The worst case rate is a quadratic Rayleigh quotient while the response is a bilinear cross moment, so no inequality between them follows from the definitions, and although every dissipative benchmark ratio lies between zero and one we do not claim that as a theorem. The ratio is a convenient normalisation for the contractive benchmark studied here, not a universal physical alignment factor, and $\Lambda^{resp}$ itself is the general output. For generators with a coherent Hamiltonian component the susceptibility is signed, the normalisation loses its bounded meaning, and the audit reports the signed value with the ratio withheld. Every predicted quantity inherits the finite probe error of order $\gamma_{probe}$ on top of the genuine second order terms in γ.

### 2.5. The validation protocol and its statistics

Throughout, measured means computed from an exact finite noise density matrix simulation of the layered circuit rather than from hardware, so every comparison is between a first order prediction and a finite noise simulation reference. Degradation is measured with common random numbers, the same parameter draws feeding the noiseless and noisy passes and shared across channels and strengths. Gradients use the parameter-shift rule with shift π/2 [3–5], and the paired estimate is $\tilde{\Delta }=1-M_{2}(\gamma )/M_{2}(0)$ over thirty retained draws with bootstrap intervals from one thousand resamples [36].

The primary correctness test of the susceptibility is direct and involves no window, no normalisation and no fit beyond a linear extrapolation. For each channel the pipeline generates one explicit array of parameter draws and passes it verbatim to both calculations, measuring the second moment at several very small per layer strengths $h\in \{2,5,10,20\}\times 10^{-5}$ on exactly the draws the estimator uses, forms the finite difference susceptibility $\Lambda_{FD}(h)=(1-M_{2}(h)/M_{2}(0))/(2h)$, and extrapolates h→0 linearly. The extrapolated limit is then compared with the generator derived $\Lambda^{resp}$, a comparison that applies unchanged to signed coherent generators, where the finite difference must reproduce a negative susceptibility. Sharing the draw ensemble is deliberate, since it isolates the first order identity itself from ensemble to ensemble sampling variation, which is assessed separately.

The same matched construction is repeated in three further directions. It is evaluated across every active parameter location at depth four, where the light cone breaks the shallow degeneracy, then on the open chain architecture, and then at ten qubits in a reduced and clearly stated setting. It is finally evaluated across a panel that varies one setting at a time, two further initialisation widths, a second hopping background and a second readout site. A draws convergence check also recomputes the susceptibility at increasing draw counts, with bootstrap interval widths and effective sample sizes. This makes the shipped ensemble sizes auditable rather than assumed. The same matched construction is repeated across every active parameter location at depth four, where the light cone breaks the shallow degeneracy, giving a parameter resolved validation map. The statistical hierarchy of the paper follows this design, the direct finite difference comparisons being primary, the finite noise intercept agreement and family error secondary, and the pooled regression of Appendix C supporting and exploratory.

The finite noise grid over γL from 0.01 to 0.3 serves a complementary purpose, locating where the first order description begins to fail. Because any finite noise ratio carries second order contamination, the empirical alignment used in the secondary analyses is the zero noise intercept of a least squares fit of $\hat{\omega }$ against γL over a per channel window in the accumulated defect $\gamma L\lambda_{coh}$. The primary window is $\gamma L\lambda_{coh}\leq 0.4$, which retains a genuine fit for every channel on the released grid, with the looser cutoff of 0.8 reported for comparison. At the tightest cutoff of 0.2 the fastest channels retain too few points for individual fits, so that cutoff enters the pooled regression only. We note candidly that the window was fixed during revision rather than in a dated analysis plan, which is why the tightening sequence is reported in full.

Validation parameters are selected by an activity preflight, which maps the mean squared noiseless gradient over the parameter grid and doubles as a light cone diagnostic. The primary parameter sits at layer two, site one, and every headline analysis uses this single location, so the experimental unit is one channel at one strength. A second location at layer one, site zero proves almost exactly degenerate with the primary one, agreeing to machine precision for most channels and to three parts in a thousand at worst. This is an architectural degeneracy of the shallow geometry, one instance of which is derived exactly in Appendix A, where the dephasing alignment at three layers takes the same value of two thirds at both insertion layers. The second location is therefore reported only as an equivalence check and never pooled as an independent setting, and a map of predicted against measured susceptibilities over genuinely non degenerate parameters remains the clearest next validation target.

The pooled comparison regresses the logarithm of the measured degradation on the logarithms of γL and of each candidate rate. The correlated control has a vanishing predicted rate, cannot enter a logarithmic fit, and is excluded identically from both models and validated separately. The null coherent control is also excluded, since it duplicates amplitude damping by construction, so the regression contains seven contributing channel clusters and every pooled statistic in the paper is computed over distinct dynamics only.

Uncertainty is assessed three ways. A channel cluster bootstrap resamples whole channels, and a leave one channel out test fits the response model on six of the seven contributing channels and predicts the seventh. Because common random numbers couple observations across channels as well as within them, a joint bootstrap over the shared draws also resamples the draw index, recomputes every second moment and refits each intercept, producing draw level intervals for every measured quantity in the archived outputs.

All computations use exact density matrix simulation at *n* = 8 qubits, the primary configuration having three layers in the single excitation sector, the depth study spanning three to six layers, and the noise grid taking γL from 0.01 to 0.3, with the direct validation probing four further strengths below 10^–3^. The response estimator uses forty draws in the main phases and twenty in the two excitation study, the measurement uses thirty draws throughout, and all randomness flows through fixed integer seeds recorded in the run manifest.

### 2.6. Software, reproducibility and computational scope

CohAlign contributes to the open source quantum software ecosystem [37] and is built on the standard scientific Python stack [38,39]. Three modules provide the sector bookkeeping, model and channels, the rates and audit, and the benchmark utilities, and their sources are materialised verbatim from the source cells of a single pipeline notebook, which keeps the released package aligned with the executed analysis. The release process includes a clean run from an empty results directory in which every table and figure is regenerated before archiving. Provenance stays linked to each experiment because every phase writes one named table, phase A through phase O, whose SHA-256 hash enters the run manifest. Each reported number therefore traces to one file of one archived run. The release is versioned under the MIT licence. The notebook runs in a smoke mode completing in minutes, a paper mode reproducing the full configuration in roughly two hours on a standard cloud processor, and a figures mode re-rendering every figure from the cached per phase outputs. Each phase caches its results, so an interrupted run resumes where it stopped.

The restricted generator uses one channel probe per pair basis element, $K=d_{r}(d_{r}-1)$ probes in all, so the probe count is polynomial in the sector pair dimension. The construction nevertheless stores a dense *K* by *K* matrix and diagonalises its Hermitian part, and every probe and propagation currently runs in the full Hilbert space of dimension $2^{n}$. Wall time and memory therefore still grow exponentially with qubit number, and a sector native implementation confining the dense linear algebra to the sector representation is the natural next step for the software.

## 3. Results

The validation is organised as a ladder, and each rung is designed to answer one question that the previous rungs leave open. We begin where the answer is known in advance, so that the machinery can be checked against exact results, and move immediately to the engineered control, where the operator and response pictures come apart. The direct finite difference validation of the susceptibility follows as the headline quantitative test, extended by a parameter resolved map across distinct locations. The later rungs widen the view, first to finite noise behaviour across the full channel family at one architecture, then across depth and into the two excitation sector, with the implementation benchmark recorded in the appendices.

### 3.1. Recovery of the operator rate and the zero alignment control

The first rung checks the machinery where the answer is known in advance. For the restricted isotropic channels the generator contracts every direction of the coherence block at a common speed, so the mode contraction rate must recover the worst case rate whatever the architecture does. At eight qubits the audit recovers this with relative error below $3\times 10^{-4}$ at the working probe, the computed rates being 1.000, 3.996, 6.648 and 7.973 for amplitude damping, dephasing, depolarising and *X* error, with the spectral anisotropy of the Hermitian part certifying isotropy itself. One remark bounds what this establishes. Isotropy guarantees that the mode rate equals the worst case rate, but not that the response reaches the same value, since the response additionally requires the contraction to be uniformly visible at every slot, a condition the depth results below show even isotropic dephasing fails slot by slot.

All twenty two shipped self tests pass here, covering trace preservation, sector preservation, frame invariance of the response decomposition, the variational bound of Equation (3) on random directions, and the analytic rates of the control generator.

The second rung is the sternest test of the distinction this paper draws. The correlated control protects coherence within each paired edge exactly while contracting cross edge coherence at nearly the full rate, the audit predicts from the generator and architecture alone that all four candidate parameters have a response of zero, and paired measurements confirm degradation below 10^–14^ at every one. The instructive case is the parameter at layer one, site zero, where the mode diagnostic returns $\omega^{mode}=0.969$, saying the channel contracts the gradient operator at almost the worst case speed, while the response prediction is zero and the measurement agrees. The damage to the operator is real but orthogonal to what the readout reads through the prepared state, so none of it appears in the derivative. A spectrum only or mode only analysis would classify this parameter as fully exposed and be wrong in the most practically relevant way. Fig 1 summarises both rungs.

**Fig 1:**
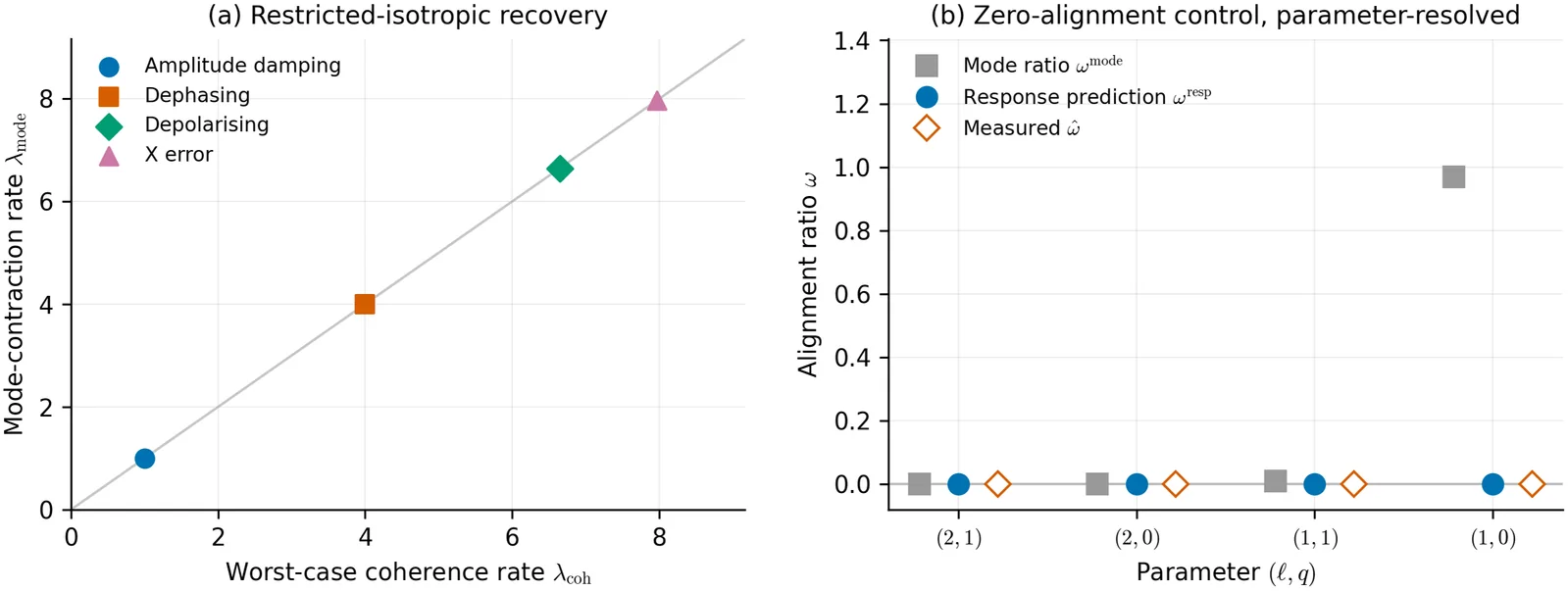
Recovery and the zero alignment control. Panel (a) shows the restricted isotropic recovery, in which the mode contraction rate for the four isotropic channels lies on the identity line against the computed worst case rate with relative error below $3\times 10^{-4}$. Panel (b) shows the control, resolved by parameter, with the three quantities drawn as separated markers so that the values at zero remain visible against the zero line. For each candidate parameter the panel gives the mode contraction ratio, the response prediction and the measured alignment. At the parameter in layer one, site zero, the mode diagnostic assigns a ratio of 0.969 while the response prediction and the measurement agree at zero.

### 3.2. Direct first order validation of the susceptibility

Having seen that the control behaves exactly as predicted, the next question is whether the computed susceptibility is numerically the true first derivative of the measured second moment. The sharpest test compares it with a separately evaluated first order response, with no window, no normalisation and no regression in between. For each channel of the suite, together with the signed pure coherent over-rotation, the pipeline measures the paired degradation at the four per layer strengths given in the Methods, forms the finite difference susceptibility $\Lambda_{FD}(h)$ on the same draw ensemble as the estimator, and extrapolates linearly to h→0. Fig 2 shows the result at eight qubits. Every dissipative channel lands on the identity line, the relative error running from $3\times 10^{-8}$ for amplitude damping, whose restricted generator the probe reproduces exactly, to $3.4\times 10^{-3}$ for the fastest probes. The residuals are precisely the order $\gamma_{probe}$ probe bias already tabulated in the convergence study, dephasing for instance returning 7.9920 against the limit of 8.0000, a relative deviation of exactly 10^–3^ at the shipped probe of 10^–3^.

**Fig 2:**
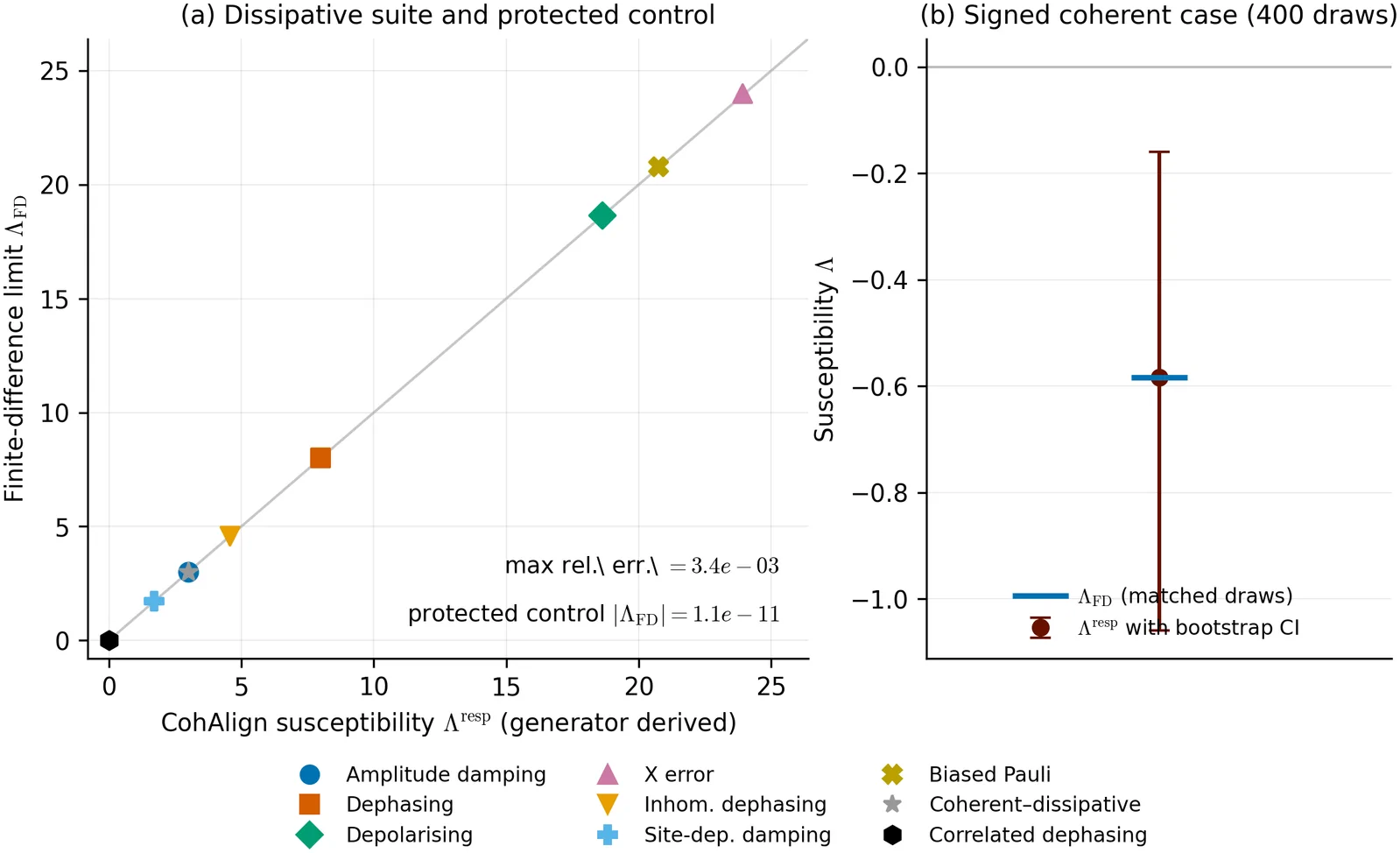
Direct first order validation of the susceptibility. Panel (a) plots the generator derived susceptibility $\Lambda^{resp}$ against the finite difference limit $\Lambda_{FD}$, obtained by extrapolating the measured degradation slope to zero noise on one shared array of parameter draws, for the dissipative suite at eight qubits, three layers and one excitation. The identity line is the first order claim, the largest relative deviation over the nonzero dissipative channels is $3.4\times 10^{-3}$, the stated probe bias, and the protected control sits at the origin with an absolute finite difference residual of $1.1\times 10^{-11}$. Panel (b) shows the signed pure coherent case on an enlarged matched ensemble of four hundred draws, the computed susceptibility with its bootstrap interval, which excludes zero, against the matched finite difference limit.

The two controls carry the conceptual weight. The protected correlated dephasing parameter, whose operator mode the channel contracts at nearly the worst case rate, returns a finite difference limit of $1.1\times 10^{-11}$, zero to numerical precision, against the predicted zero. The inequivalence of contraction and response is therefore confirmed by a direct derivative of the measured second moment, and not merely by the finite noise reference points.

The signed site dependent *Z* over-rotation is a harder case, because its per draw contributions cancel heavily and a small matched ensemble cannot by itself establish the sign of the population susceptibility. The pipeline therefore evaluates this channel on an enlarged matched ensemble of four hundred draws, one explicit draw array passed verbatim to both the audit and the reference. On that ensemble the computed susceptibility is −0.584 with a bootstrap interval from −1.06 to −0.16, which excludes zero, and the finite difference limit is −0.584, agreement to $3.5\times 10^{-5}$. The first order enhancement of the ensemble gradient second moment, which no contraction based diagnostic can express, is therefore established with a stable sign and measured directly. The negative slope of the second moment ratio confirms that this coherent perturbation strengthens the second moment at first order rather than degrading it.

The same identity is then tested where the phrase parameter resolved earns its meaning, across many genuinely distinct locations in one circuit. At depth four, where the growing light cone breaks the shallow geometry degeneracy, the activity preflight admits nine locations. The audit and the matched finite difference are evaluated at every one for five channels, dephasing, the correlated control, amplitude damping, inhomogeneous dephasing and the covariance breaking biased Pauli probe. Seven locations per channel support the matched measurement, while two marginal locations per channel fall below the measurement floor and are recorded as such in the released table rather than silently dropped. Fig 3 shows the thirty five resulting measurable parameter and channel pairs. The susceptibilities genuinely vary, from 2.73 to 27.4, a factor of ten, the locations falling into a small number of architectural symmetry classes with distinct values across classes and across channels. Every point lands on the identity line at its expected bias, from $1.9\times 10^{-8}$ for amplitude damping, whose restricted generator the probe reproduces exactly, through $1.0\times 10^{-3}$ for the dephasing type channels to $3.0\times 10^{-3}$ for the covariance breaking probe.

**Fig 3:**
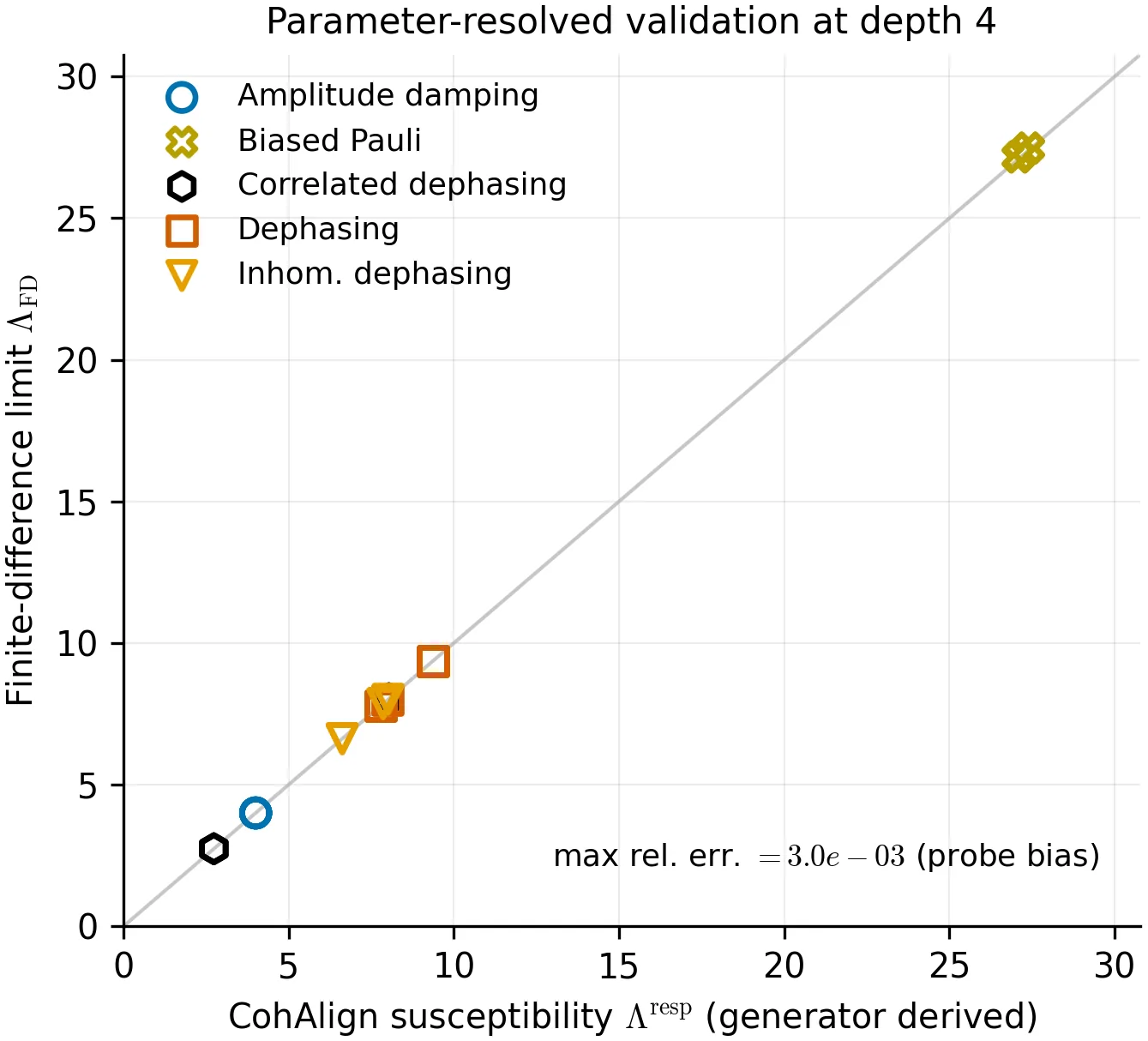
The parameter resolved validation map. The generator derived susceptibility is plotted against the matched finite difference limit for every active parameter location at eight qubits and depth four, for the five channels indicated. Each channel contributes seven measurable locations, values differing across locations and channels by a factor of ten, and every point agrees with its finite difference at its expected probe bias, two further marginal locations per channel being recorded at the measurement floor in the released table.

The map also exposes an interplay no scalar could carry, since the locations that dephasing degrades fastest, at 9.36, are precisely the ones the correlated control leaves most protected, at 2.73.

### 3.3. The identity across architecture, system size and settings

The strength of a direct identity is that it can simply be evaluated somewhere else, so the same audit against matched finite difference comparison is repeated in three further directions. On the open chain, the same gate set with the cycle closing edge removed, dephasing lands at the probe bias of $1.0\times 10^{-3}$ and amplitude damping is reproduced to $9\times 10^{-9}$. The correlated control transfers its sharpest behaviour, the audit predicting a response of exactly zero at the selected chain parameter and the finite difference confirming zero with an absolute residual of $4\times 10^{-11}$. At ten qubits, in a reduced and clearly stated setting of twenty draws and a two point strength grid, dephasing again lands at the probe bias and amplitude damping at $1.7\times 10^{-8}$. Fig 4 collects these points on one identity plot.

**Fig 4:**
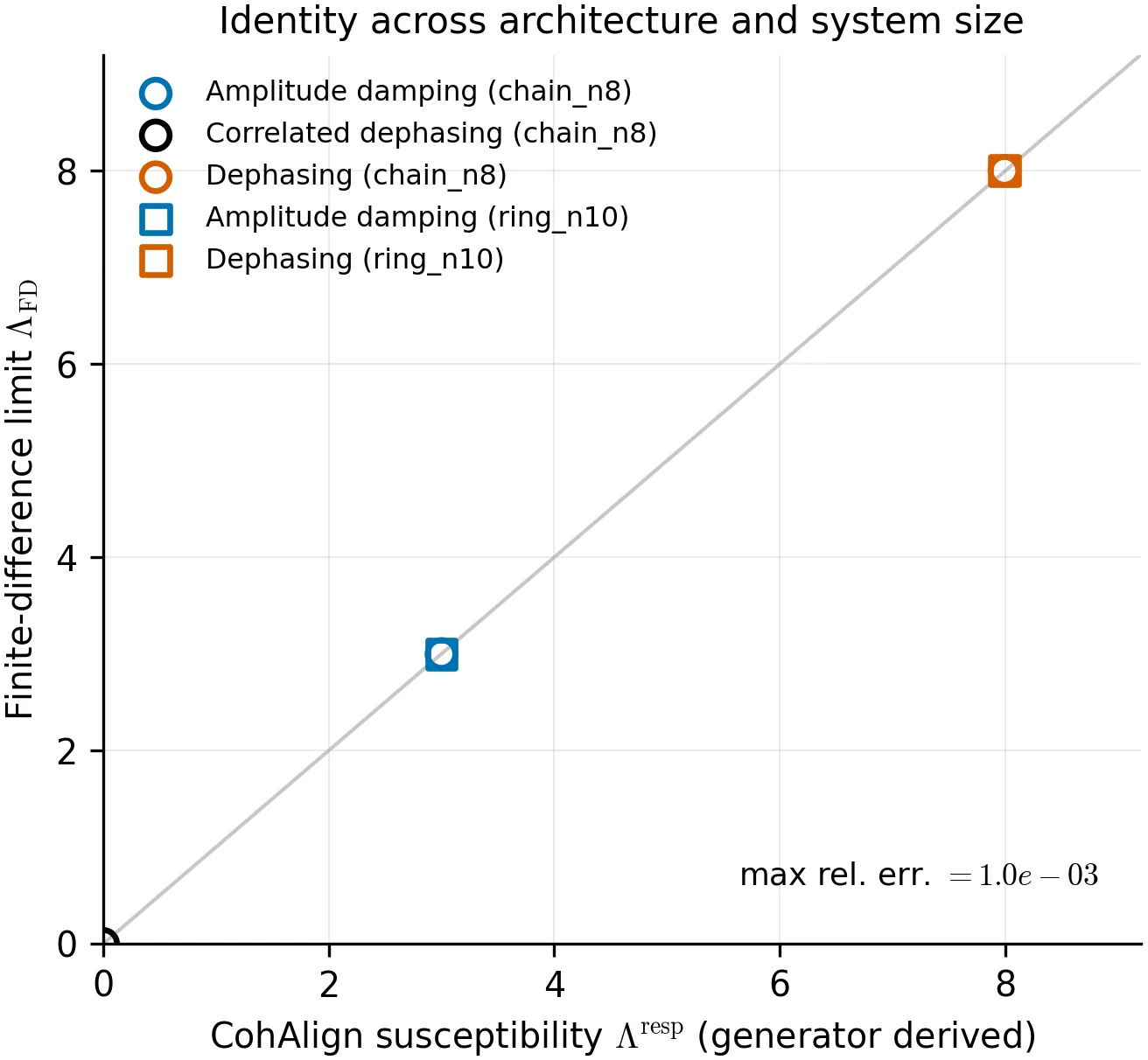
The identity across architecture and system size. The generator derived susceptibility is plotted against the matched finite difference limit for the open chain at eight qubits and the ring at ten qubits, for the channels indicated. Every point lands on the identity line at its expected bias, and the chain’s correlated control point sits at the origin with an absolute residual of $4\times 10^{-11}$.

The settings panel then varies one ingredient at a time at the primary geometry, two further initialisation widths of 0.02 and 0.1, a second hopping background, and a second readout site. The readout variation is evaluated on the delocalised sector input, since with the localised input an off cone readout at shallow depth has every gradient at numerical zero, an observation that is itself a light cone confirmation. Every measurable row agrees with its matched finite difference to at most $3.5\times 10^{-4}$ at the single strength used, and the readout variation is not a trivial repetition, the dephasing susceptibility moving from 7.99 to 7.38 when the readout moves, exactly the readout dependence the construction claims. A separate draws convergence check recomputes the susceptibility at ten, twenty, forty and eighty draws, the estimate unchanged to all reported digits while the bootstrap interval narrows roughly as the square root of the draw count, which is why forty draws suffice for the headline phases. The signed coherent case, whose per draw contributions cancel heavily, is the one place the enlarged ensemble of four hundred draws is needed.

### 3.4. Alignment across the channel family

With the susceptibility itself validated, the next rung widens the view to finite noise behaviour across the full benchmark. Table 3 reports the audit at the primary parameter, every rate computed at generator level, together with the measured alignment, arranged with the six phase covariant dissipative channels above the rule and the three controls below it. The suite is a designed benchmark rather than nine independent draws from a channel population. The coherent dissipative row is a null control identical to amplitude damping by construction, so eight distinct channels contribute, and the null row is excluded from every family statistic.

**Table 3:** The generator level audit at eight qubits, three layers and one excitation, with measured alignment. Rates are computed spectrally for every channel, and $\hat{\omega }$ is the zero noise intercept over the accumulated defect window $\gamma L\lambda_{coh}\leq 0.4$ at the primary parameter, layer two, site one. The upper block contains the phase covariant dissipative channels, and the lower block contains the controls. The coherent dissipative row is a null control that is identical to amplitude damping by construction, so it is an invariance check rather than an independent validation case, and eight distinct channels contribute to the family statistics. The typical sector rate $\lambda_{coh}^{typ}$ is omitted here as it plays no role in the central argument and is available in the released outputs.

Channel	$\lambda_{coh}^{worst}$	$\lambda_{mode}$	$\omega^{mode}$	$\omega^{resp}$	$\hat{\omega }$
Amplitude damping	1.0000	1.0000	1.0000	1.0000	0.9962
Dephasing	3.9960	3.9960	1.0000	0.6667	0.6635
Depolarising	6.6476	6.6476	1.0000	0.9335	0.9235
Inhom. dephasing	5.7061	2.2844	0.4004	0.2669	0.2669
Site-dep. damping	1.4286	0.5714	0.4000	0.4000	0.3997
Correlated dephasing	3.9960	0.0000	0.0000	0.0000	0.0000
*X* error	7.9731	7.9711	0.9997	1.0000	0.9820
Biased Pauli	7.1776	7.1773	1.0000	0.9631	0.9501
Coherent–dissipative	1.0000	1.0000	1.0000	1.0000	0.9962

At the primary parameter the predicted ratio matches the defect windowed intercept with root mean square error 0.009 across the eight distinct channels. Raw extraction without the intercept gives 0.075, with residuals uniformly negative and growing with the channel rate, the signature of second order contamination rather than estimator error. Every measured intercept carries a joint draw level bootstrap interval narrower than 10^–3^ in exact simulation under common random numbers, as is the interval on the family root mean square error, so the dominant uncertainty is the probe bias and the genuine higher order terms rather than sampling.

Split by compliance class, the six phase covariant dissipative channels agree at 0.005 and the two covariance breaking probes at 0.016, the *X* error channel being the largest single residual at 0.018. This is the ordering one hopes to see, since the probes were included to stress the machinery outside its cleanest regime and part of their response reflects sector leakage. The null coherent control audits identically to amplitude damping, exactly as its in-sector triviality requires, and the mode ratio of the correlated control in the table refers to the primary parameter, whereas the 0.969 quoted earlier belongs to layer one, site zero, so the two statements are compatible.

The first panel of Fig 5 shows the prediction tracking the measured degradation across the entire grid, and the per channel alignment scatter is released with the archived figures. The second panel plots the ratio of measurement to prediction against the predicted degradation $2\gamma \Lambda^{resp}$ itself, the most response matched horizontal variable, the median falling smoothly from 0.98 at the weakest noise to 0.52 at the strongest as genuine higher order effects accumulate. We report this departure rather than fit it away, because it marks the boundary of the claimed regime, and it supports a practical rule. On the released grid the measured degradation stays within four percent of the prediction wherever the predicted degradation $2\gamma \Lambda^{resp}$ is below 0.1, and within ten percent below 0.2. A user who keeps the predicted degradation below those thresholds can therefore trust the first order number to that accuracy in configurations like the one tested, while larger predicted degradations call for the finite noise reference the pipeline also provides.

**Fig 5:**
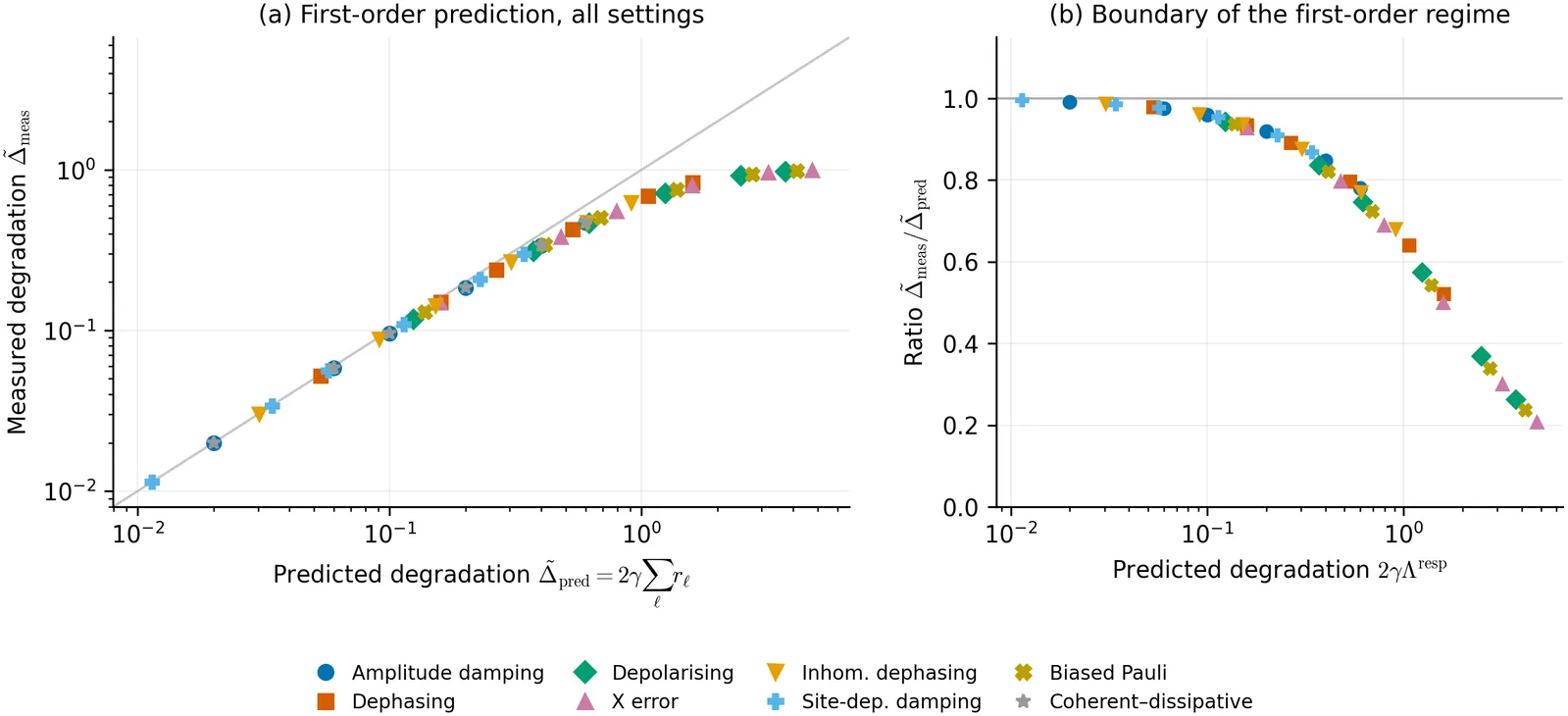
The first order prediction and the boundary of its regime. Panel (a) plots predicted against measured degradation for every channel and strength at the primary parameter on logarithmic axes, the departures at large predicted degradation being the expected higher order corrections. Panel (b) plots the ratio of measured to predicted degradation against the predicted degradation $2\gamma \Lambda^{resp}$ itself, the horizontal line marking the first order prediction, so the smooth fall of the ratio locates the boundary of the perturbative regime without any fitted regression. The null duplicate is excluded from both panels.

As a secondary finite noise analysis, replacing the worst case rate with the response rate substantially improved the pooled organisation of the data, the coefficient of determination at the primary window rising from 0.83 to 0.998, and the improvement strengthened monotonically as the accumulated defect window was tightened. The full regression table, the channel cluster bootstrap, the joint draw level interval, and the leave one channel out test are recorded in Appendix C, since the direct finite difference comparison above carries the quantitative claim and the pooled fit is a consistency check rather than primary evidence.

### 3.5. Depth, the higher charge sector and computational cost

The next rung asks whether the picture survives when the architecture changes. The layer resolved structure of the response yields predictions that no scalar could express, and for dephasing the final noise slot contributes nothing, for a reason that repays statement. The derivative operator reaching the final slot is contracted by the channel, but the resulting change has zero overlap with the readout functional there, so none of the contraction appears in the derivative. Combined with the layer resolved values of the remaining slots this gives two thirds at three layers and one half at four, and Appendix A records both the exact final slot argument and the slot structure behind each total. The measured alignments, extracted at the weakest grid strength, come in at 0.652 and 0.492, and across the depth study from three to six layers the median absolute difference between predicted and measured alignment is 0.009, as Fig 6 shows.

**Fig 6:**
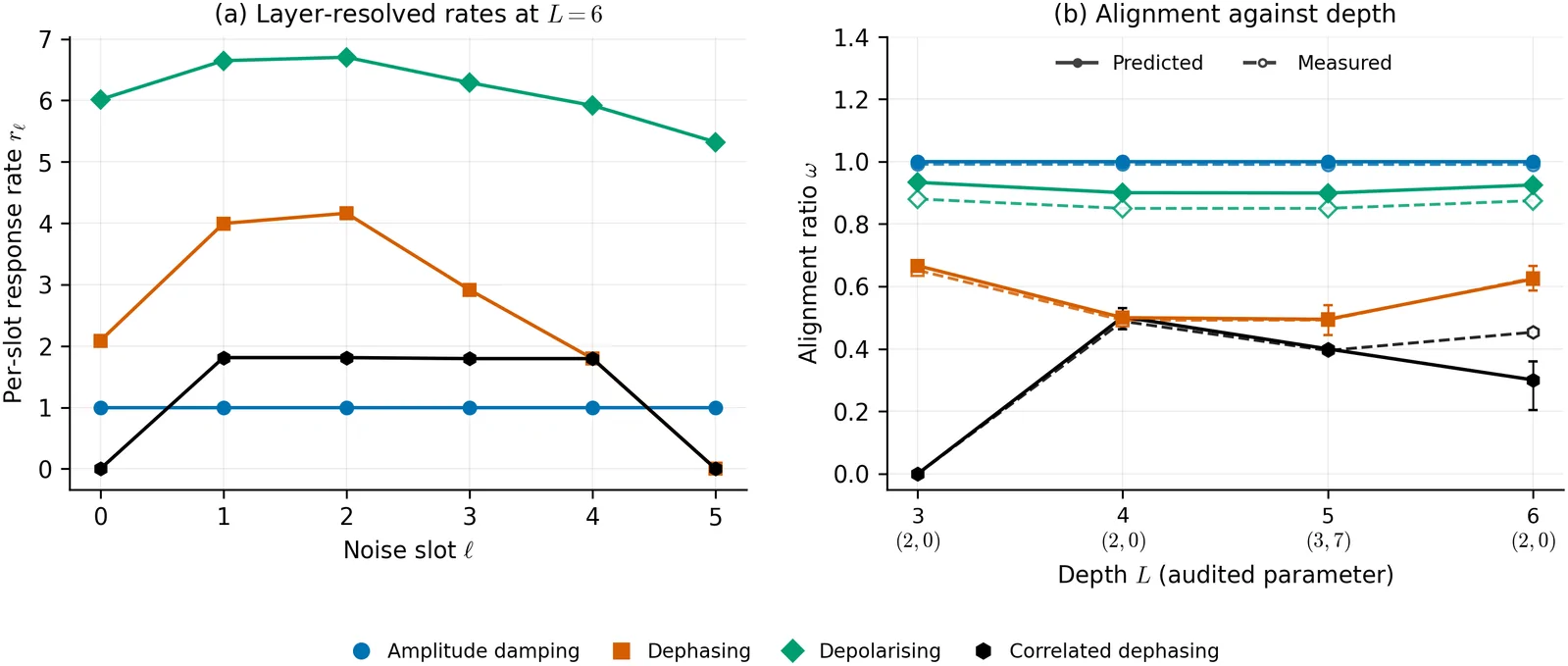
Layer resolved rates and the depth study. Panel (a) shows the per slot response rates at the deepest audited architecture. Panel (b) shows predicted alignment as solid lines with bootstrap intervals and measured alignment as dashed lines against depth, including the closed form dephasing values of two thirds and one half and the switching on of the control’s exposure once the light cone crosses its protected pairing, with the audited parameter printed beneath each depth.

One caveat is to be noted here. The activity preflight selects the most responsive parameter at each depth, so the comparison is an activity selected study of architecture and depth together, with the audited location printed beneath each depth in the figure and the exact values belonging to those locations.

The control adds a further lesson at depth, its protection being a property of the shallow light cone. Once the growing cone carries the gradient mode across the paired edges the alignment switches on, with 0.503 predicted against 0.488 measured at four layers and 0.400 against 0.395 at five. At six layers, both prediction and measurement become sample limited, the weight concentration diagnostic indicating roughly eleven of forty draws carrying the estimate, and this deepest point is recorded as an open stress case in Appendix B rather than as part of the main evidence.

The final scientific rung moves to the two excitation sector, the sternest family level test because the coherence block grows to 756 dimensions at eight qubits and scalar summaries are least likely to suffice. One architectural fact required a change of input. At the primary shallow geometry the localised basis state carries no trainable phase response at all for this readout, the preflight returning a map at the numerical floor, and the software raises an explicit error here rather than silently selecting noise. The inactivity belongs to that specific geometry rather than to higher sectors in general, so the validation uses a fixed delocalised input, drawn once with a recorded seed as a normalised complex Gaussian superposition over the sector basis and shared across channels.

This phase uses twenty estimator draws rather than forty, a difference we disclose because the response is explicitly state dependent. For the same reason the pipeline repeats the audit across three delocalised input seeds, the predictions varying by about 0.04, for dephasing between 0.148 and 0.192, comparable to the sector root mean square error, so the input dependence is quantified rather than assumed away. Across the eight distinct channels, the null control again excluded, the predicted alignment matches measurement with root mean square error 0.037, as Fig 7 shows, with measurement to prediction ratios between 0.92 and 0.99 at the weakest strength tabulated in the released outputs. The sector also resolves the channels in ways the single excitation study cannot. Dephasing collapses to 0.153 against a measured 0.151, amplitude damping stays at unity against 0.982, and the correlated control is no longer protected at all, predicted 0.1531 against measured 0.1517.

**Fig 7:**
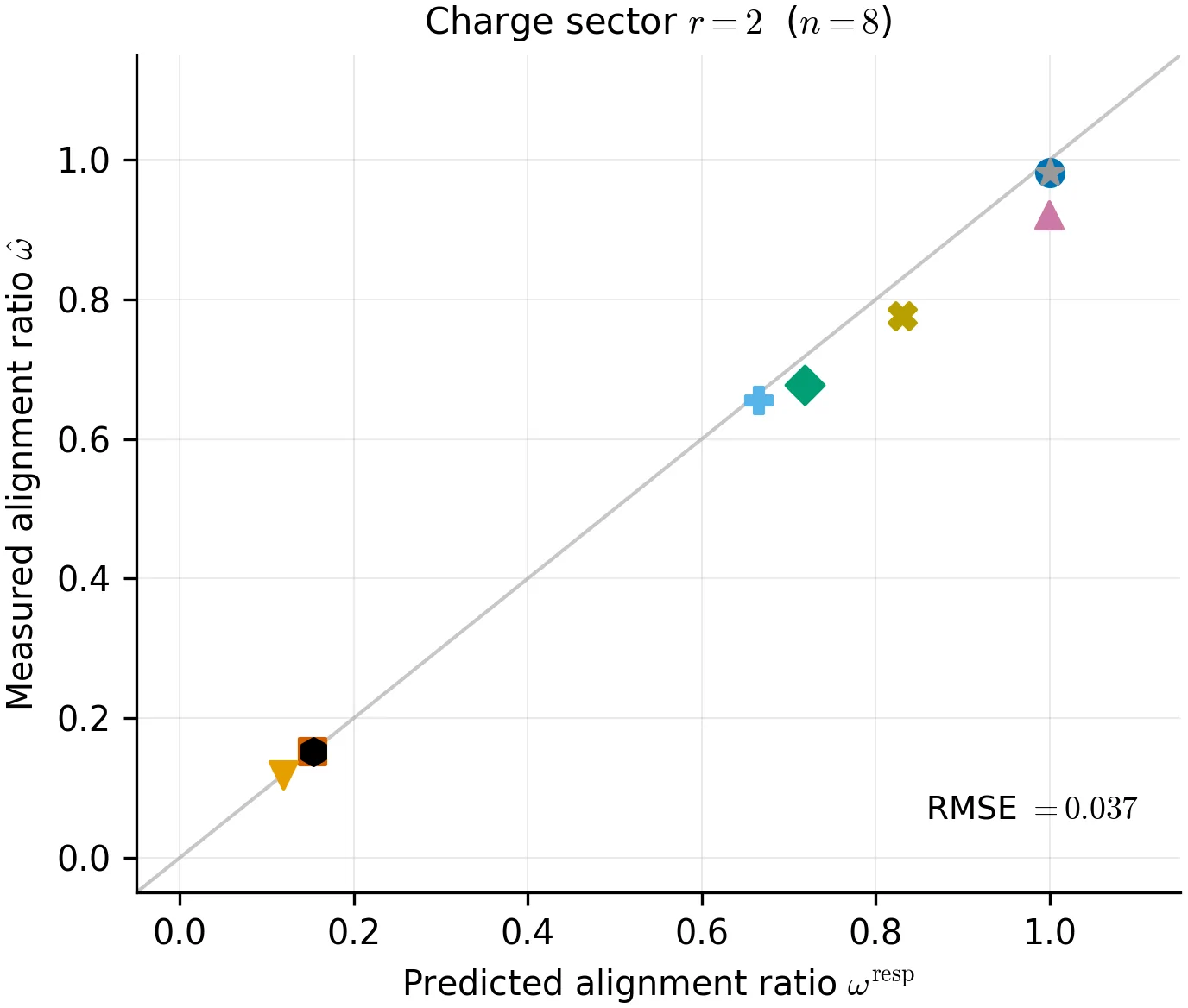
The higher sector transfer test. Predicted alignment is plotted against measurement in the two excitation sector, with the correlated control correctly predicted to be unprotected. The null duplicate is shown for completeness but excluded from the annotated root mean square error.

The mechanism deserves further clarification, since it is a property of the tested configuration rather than of the sector as a whole. For the selected delocalised input, readout and parameter, the visible derivative response has nonzero support on cross edge coherence directions, so the protected within edge subspace no longer exhausts the response. The result should be read as a bounded transfer test for one selected state, with the input seed sweep quantifying how much that state matters.

The final rung concerns cost, reported as an implementation benchmark of the released code rather than as a scientific claim. The pipeline ships a matched timing benchmark in which the generator construction, the complete audit and the equivalent finite noise sweep are each timed as medians of three repetitions with spreads recorded, the restricted generator being built once per channel and reused through the channel context. Fig 8 in Appendix B shows the scaling with system size from the archived run. The structural property worth noting is not any single number. The audit involves no layered finite noise circuit sweep and no grid of noise strengths, so auditing every readout visible parameter costs a small multiple of one audit, while the equivalent simulation campaign multiplies parameters, grid points and draws together. The processor, core count, operating system and package versions are recorded in the run manifest.

**Fig 8:**
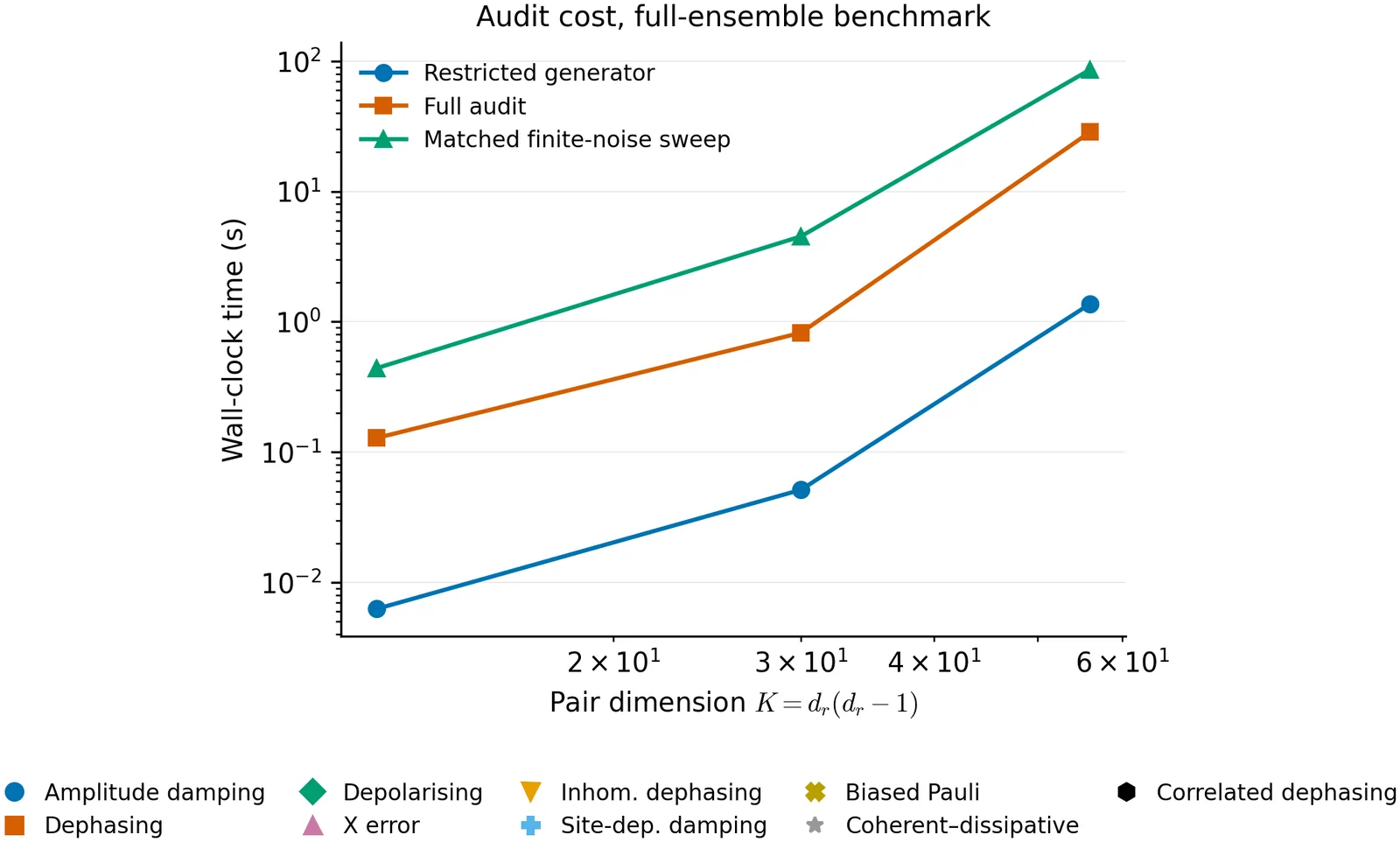
The implementation cost benchmark. Wall clock cost of the generator construction, the full ensemble audit and the matched finite noise sweep against system size, all timings medians of three repetitions at full settings with spreads recorded in the released outputs. These timings are machine and implementation dependent and form no part of the scientific validation.

### 3.6. From prediction to decision

To provide further validation, we present two worked applications. In the first, every measurable location in the depth-four geometry is audited under both dephasing and correlated control, then measured once at a working strength of 0.01 on the matched ensemble. Table 4 condenses the fourteen resulting rows to the six symmetry-class representatives. The measured retentions follow exactly the ranking predicted by the audit. The preferred placement also reverses when the noise model changes. The location that degrades most rapidly under dephasing, retaining 0.829, is the one best protected by correlated control, retaining 0.949. Across the table, the prediction-to-measurement ratios range from 0.92 to 0.93 for predicted degradations near 0.19, reaffirming the stated trust rule on new data.

**Table 4:** Noise aware parameter placement at depth four. Each measurable location is audited and then measured once at working strength γ=0.01 on the matched ensemble, retention being one minus the degradation of the ensemble gradient second moment. The seven locations fall into symmetry classes and one representative per class is shown. The best placement under dephasing is the worst class under the correlated control and the reverse, so the decision flips with the noise model.

Channel	Location	$\Lambda^{resp}$	Predicted retention	Measured retention
Dephasing	(1,0)	9.359	0.813	0.829
Dephasing	(2,0)	7.992	0.840	0.851
Dephasing	(2,7)	7.778	0.844	0.854
Correlated control	(1,0)	2.734	0.945	0.949
Correlated control	(2,0)	8.032	0.839	0.851
Correlated control	(2,7)	7.992	0.840	0.851

In the second application, a composite channel applies dephasing and amplitude damping at each layer. Their strengths are in the ratio 0.7 to 0.3. The channel is built using the custom channel interface. A user can halve one component and must choose which to reduce. The audited susceptibilities are 9.359 for dephasing and 4.000 for amplitude damping. The additive first-order prediction for the composite baseline degradation is 0.155. The measured value is 0.144. Halving dephasing reduces the measured degradation by 0.058. Halving damping reduces it by only 0.010. These measurements confirm the recommendation by the audit. Both tables can be regenerated using a standalone applications notebook released with the repository.

## 4. Discussion

The practical value of a computable susceptibility is that noise awareness moves from description to prediction at design time. Because it is resolved by parameter, it distinguishes individual parameters and not merely whole channels. As the control illustrates, the same channel that leaves the shallow network untouched exposes it once the light cone crosses its protected pairing. Where a noiseless symmetry audit [26,27] or a channel level diagnostic [28] establishes whether architecture and channel respect the symmetry, CohAlign establishes how fast the surviving readout visible coherence is destroyed.

Conceptually, the ladder separates two quantities that are easily conflated. The mode contraction rate identifies the channel and mode mechanism and is independent of the prepared state, whereas the response susceptibility evaluates the complete first order effect on the measured derivative, including the state and slot dependent terms that decide whether the contraction is actually seen. In our control, the two differ by nearly the full worst case rate. In isotropic dephasing they differ slot by slot, and any analysis identifying them implicitly assumes the contraction is uniformly read out across the slots, a condition that fails in simple and physically natural situations. Nor should the empirical ordering in our tables, where the mode ratio never falls below the response ratio, be read as a law. The response is bilinear and the mode rate quadratic, so no inequality follows from the definitions, and for coherent generators the response is signed, and the ratio loses its meaning.

It is also important to bound the predicted quantity. CohAlign predicts the decay of the ensemble second moment of a chosen gradient under a specified prior, both reported rates inheriting a dependence on that prior. The audit therefore does not by itself predict the gradient at a trained parameter vector, optimiser convergence, accuracy or shot complexity, and connecting the second moment to those downstream quantities is a separate modelling step. More broadly, the theme of preserving the task relevant part of a signal under noise recurs across domains, for example in perturbation aware representations for anomalous sound detection [40], and the response susceptibility is the form this concern takes for gradients in noisy quantum circuits.

Two further limitations can be pointed out. The one unresolved measurement, the six layer correlated control recorded in Appendix B, indicates where the estimator can become fragile, deep circuits in which a few draws carry most of the estimator weight. A low effective sample size in the report is the user visible warning to treat such a prediction with caution. It should also be clear that the nine channels form a designed benchmark rather than reconstructed device noise, and that real devices add gate and layer dependence on top of any fixed channel model. This is a concern with a long engineering history in classical hardware, where reliability under device and supply variation is designed for explicitly [41]. Hardware applicability therefore runs through the characterised noise conversion pathway described above rather than being claimed here.

A further consideration concerns expressivity. Restricting attention to a fixed charge sector narrows the accessible Hilbert space, and the one and two excitation sectors validated here are polynomially small. Hence, the symmetry constraint trades expressivity for structure in the way studied in the equivariant learning literature [15,17,18]. This trade-off is not intrinsic to the symmetry. Under the same charge conservation, the half-filling sector grows essentially as fast as the full space. A charge-conserving architecture can therefore remain exponentially expressive. The susceptibility construction is also not limited to small sectors. The restricted generator is defined separately for each sector and applies unchanged to exponentially large sectors. The previously stated cost caveat still applies as the probe count grows with the sector-pair dimension.

The predictor is first order, the smooth departure at large accumulated defect marking its regime boundary, and every prediction inherits the order $\gamma_{probe}$ probe error, removable for the reference channels through the tabulated probe convergence. The validation uses one architecture family at eight qubits, one hopping background, one readout, one initialisation width and, in the higher sector, delocalised inputs whose seed dependence the stability check quantifies. The nine channels form a designed benchmark suite rather than a random sample of device noise, with two dissipative channels producing identical results by construction.

The family wide comparison contains one unique active parameter location at the shallow depth, but the parameter resolved map at depth four validates the susceptibility directly across seven distinct locations for five channels. The same identity holds on the open chain, at ten qubits and across the settings panel. The phrase parameter resolved is therefore backed by per parameter finite differences across thirty five pairs rather than by the control alone. Wider maps over more channels and architectures, together with sweeps over background seeds, widths, readouts and randomly generated covariant generators, are stated as future work.

The six layer control point remains open at the shipped ensembles and is recorded, with its numbers, as a deep circuit stress case in Appendix B, outside the principal evidence, which the released pipeline can resolve with enlarged ensembles and which we report rather than explain away. Finally, a tomographically reconstructed channel is a fixed map rather than a calibrated family with an identity limit, so consuming gate set tomography [42] requires an error generator relative to the ideal operation, a branch choice for the channel logarithm and a Markovianity check. Real devices also exhibit gate and layer dependent noise where the present implementation applies one channel uniformly after every layer.

## 5. Conclusions

In this paper, we have presented CohAlign, an open source toolkit that computes the first order susceptibility of the initialisation ensemble second moment of a specified parameter gradient, resolved by parameter and by noise slot. The input is a classical description of an equivariant quantum neural network, a differentiable noise model, a prepared state, a readout and an initialisation prior, and no finite noise circuit sweep and no measured degradation data are required. Noise awareness thereby moves from description following a calculation available at design time.

The validation established two things. The engineered control shows that this quantity cannot be inferred from operator level contraction alone, since a channel that contracts the gradient carrying operator at nearly the full worst case rate leaves the measured response untouched, exactly as the toolkit predicts from the generator and architecture. Direct finite difference simulations then validate the first order calculation itself in the tested model and sector configurations, including a signed pure coherent case established on an enlarged matched ensemble and a parameter resolved map across distinct locations at depth four. The same identity transfers to an open chain architecture, to ten qubits and across varied initialisation widths, hopping backgrounds and readout sites.

These conclusions apply only to the small-system exact-simulation regime in which they were established, i.e., two topologies of the brickwork family at eight qubits and a reduced check at ten. The current implementation still propagates in the full Hilbert space, so wall time and memory grow exponentially with the qubit number. A sector native implementation and a principled conversion layer to experimentally characterised noise are considered future work.

### Appendix A. The ensemble identity and test battery

Squaring the first order expansion of Equation (6) and averaging over the prior gives $M_{2}(\gamma )=𝔼[g_{0}^{2}]+2\gamma \;𝔼[g_{0}\sum_{\ell }h_{\ell }]+O(\gamma^{2})$, and dividing by *M*_2_(0) yields Equation (7) directly. The form also shows why a plain mean of per draw ratios can fail. When the derivative varies strongly across the ensemble, a per draw ratio divides by small numbers and its unweighted mean no longer represents the measured ratio of second moments. The cross moment instead coincides with the derivative squared weighted mean of the ratios wherever those are defined and remains meaningful everywhere.

The exact dephasing ratios follow from an orthogonality argument. At the final noise slot, the backward evolved readout is $O_{B}$ itself, diagonal in the computational basis, while the dephasing generator maps any operator into a purely off-diagonal one, so the trace pairing vanishes identically for every draw, not merely on average. At three layers, the pipeline further verifies, slot by slot, that both earlier slots pair the functional with modes contracting at the uniform rate $\lambda_{coh}$, so the alignment at that depth is exactly two thirds. We caution that this does not extend to a general law of the form (L−1)/L. At four layers, the layer resolved values show the final slot at zero and two slots at the full rate, while the response of one earlier slot is numerically cancelled by the architecture, so the total is one half rather than three quarters. We therefore present two thirds and one half as computed layer resolved results for the stated parameter and readout geometry, with only the final slot statement claimed as a general exact property.

The protection of the correlated control combines a fixed point property with light cone geometry. A single excitation coherence whose two sites lie within one protected edge sees equal eigenvalues under every two site phase flip and is an exact fixed point of the channel. At three layers, the backward light cone confines the visible part of the gradient mode to such directions for every candidate parameter, which the pipeline verifies numerically to below 10^–14^.

The pipeline ships a battery of twenty two self tests that run before any validation phase. They verify trace preservation of all channels to below one part in 10^15^, sector preservation of the noiseless circuit to below one part in 10^10^, and frame invariance of the response decomposition to better than one part in 10^8^. They also verify the variational property of Equation (3) on random directions and the analytic rates of the control generator. The activity preflight additionally raises an explicit error on inputs that carry no trainable phase response.

### Appendix B. Software interface, stress case and cost benchmark

One deep circuit stress case is recorded here, outside the principal evidence. At six layers, the correlated control gives a predicted alignment of 0.301 with interval from 0.204 to 0.359 against a measured 0.453, the weight concentration diagnostic indicating that roughly eleven of the forty estimator draws carry the estimate at that depth. The measured value therefore lies outside the prediction interval, and we treat the point as an unresolved deep circuit discrepancy at the current sampling budget, its cause not established, rather than as evidence for or against the estimator. The released pipeline contains everything needed to resolve it, namely the division free estimator, enlargeable ensembles, the explicit draw arrays and the direct finite difference reference.

The package comprises three modules importing only the standard scientific Python stack [38,39]. The core module provides the sector bookkeeping, the brickwork model, the channels and the trace checks. The rates module provides the restricted generator, the rates of Equations (3) and (5), the response of Equation (7) and the single call audit, and the benchmark module provides the preflight, the common random number measurement and the regression utilities, the pipeline notebook composing these into the phases of Section 2.6.

A user audits a custom channel by subclassing the channel base class with a method that applies the channel to an arbitrary matrix at a given strength, preparing a channel context once, and calling the audit for each parameter of interest. Correlated channels override the application method directly rather than supplying single qubit Kraus operators. The returned report contains the worst case and typical rates, the mode contraction ratio, the signed response susceptibility with its bootstrap interval, the normalised alignment when the generator is contractive, the per slot resolution, the weight concentration diagnostic and the effective bootstrap settings. A flag marks pure coherent generators, for which the signed response is reported and the ratio withheld. These keys are the stable public interface of the release, historical names remaining available as deprecated aliases, and the released repository contains the full worked example as an executable quick start.

### Appendix C. Pooled regression and secondary statistics

The regression level comparison asks whether the response rate can replace the worst case rate as the organising variable of a pooled degradation law, and Table 5 reports the fits over the tightening windows at the primary parameter, one observation per channel and strength. The pattern is what a correct first order predictor must produce. As the window tightens from 0.8 through 0.4 to 0.2 the response model improves monotonically, the coefficient of determination moving from 0.993 through 0.998 to 0.999 and the residual error falling from 0.094 to 0.023. The exponents on noise strength and rate reach 0.92 and 0.91 at the primary window. The worst case model does not improve at all, moving between 0.85 and 0.76 across the cutoffs, its residual variance being the alignment structure it cannot express.

**Table 5:** The pooled predictor comparison over tightening accumulated defect windows. Each fit regresses the logarithm of the measured degradation on the logarithm of γL and the logarithm of the candidate rate, at the primary parameter. The protected control and the null duplicate are excluded identically from both fits, so seven channel clusters contribute. The sample size *n* counts one observation per channel and strength.

Window	Model	*R* ^2^	RMSE	*n*	Rate exp.
0.8	$\lambda_{coh}$	0.851	0.420	33	0.913
0.8	Response rate	0.993	0.094	33	0.864
0.4	$\lambda_{coh}$	0.828	0.429	27	0.947
0.4	Response rate	0.998	0.048	27	0.912
0.2	$\lambda_{coh}$	0.756	0.449	18	0.901
0.2	Response rate	0.999	0.023	18	0.954

A channel cluster bootstrap places the response model’s coefficient of determination between 0.997 and 0.999. The cluster interval on the difference between the models runs from just below zero to 0.28, wide because only seven clusters feed it, a width we report plainly and which counsels reading the comparison through the monotone tightening pattern rather than any single interval. The complementary draw level joint bootstrap places the difference at 0.17 with an interval narrower than 10^–3^, sampling error being negligible in exact simulation. The leave one channel out test gives pooled held out root mean square log error 0.057, with the worst single channel the *X* error probe at 0.083.
